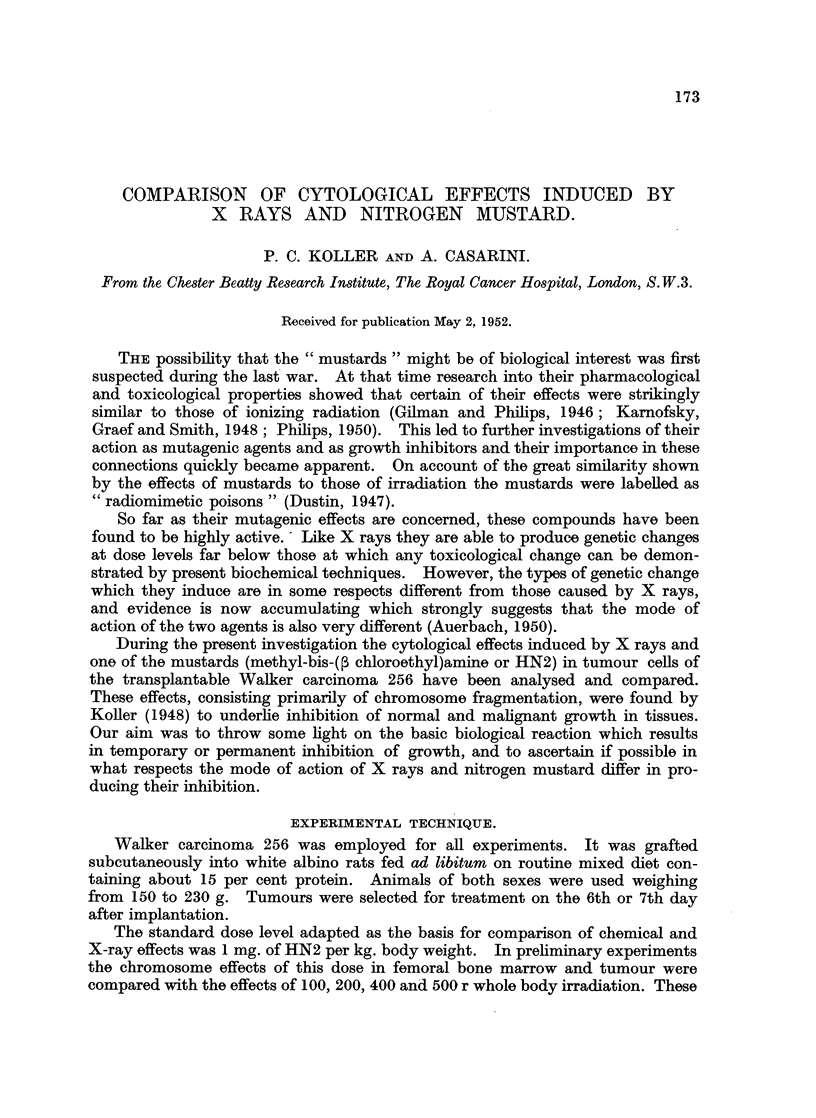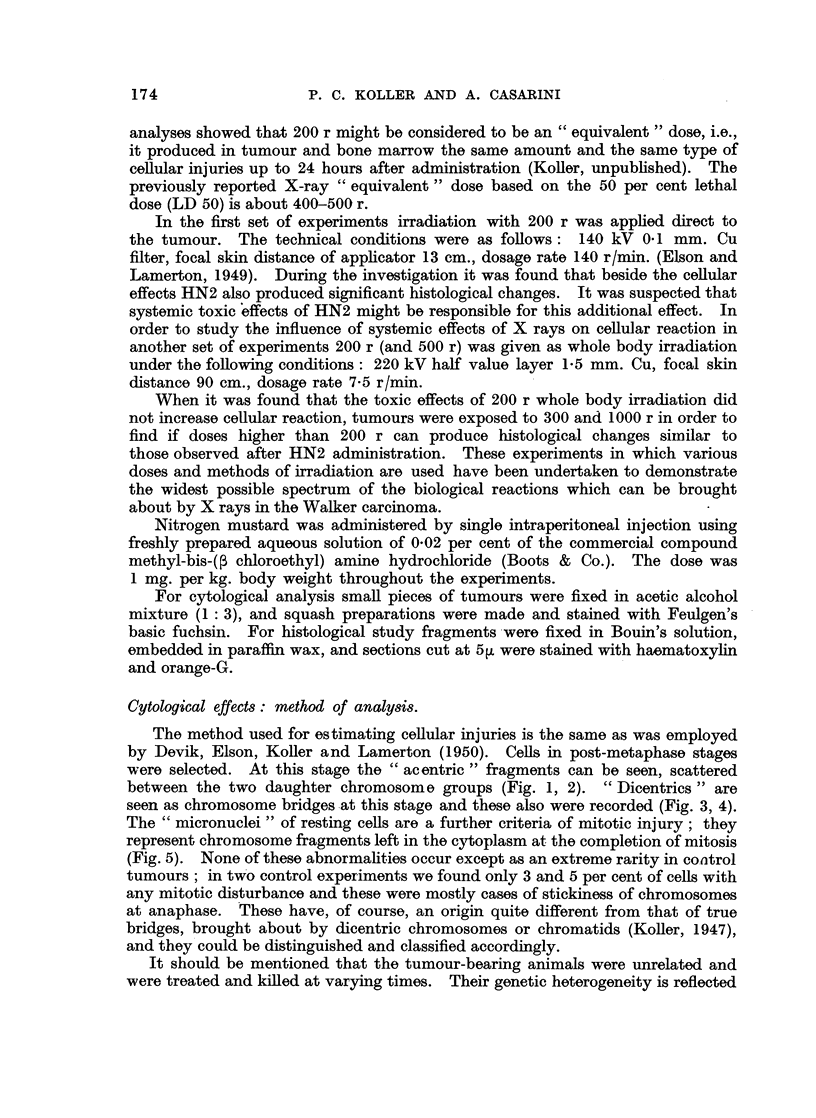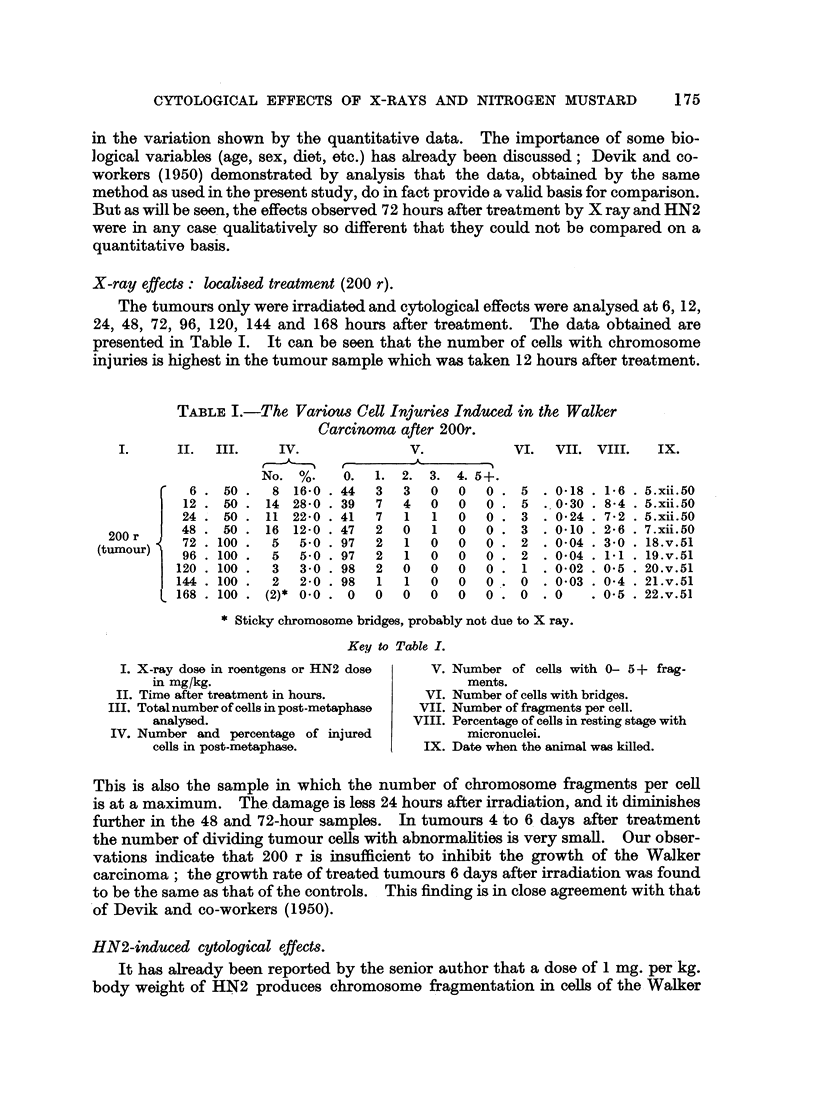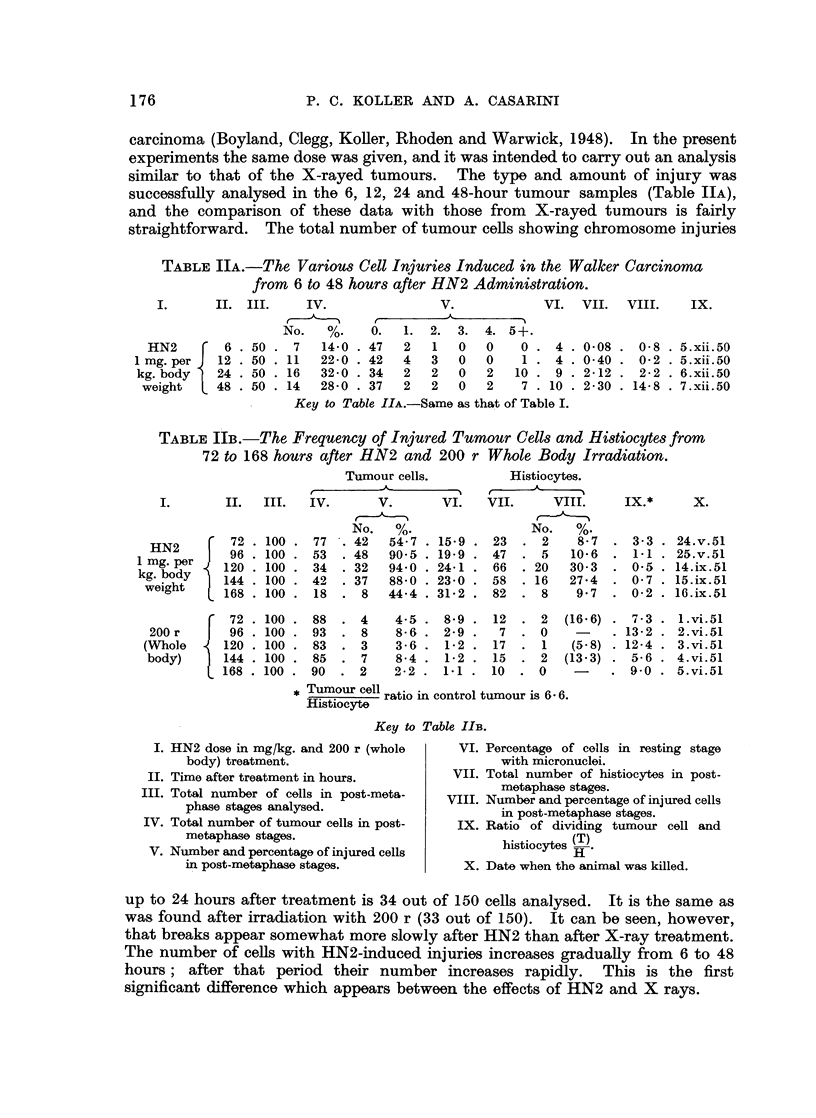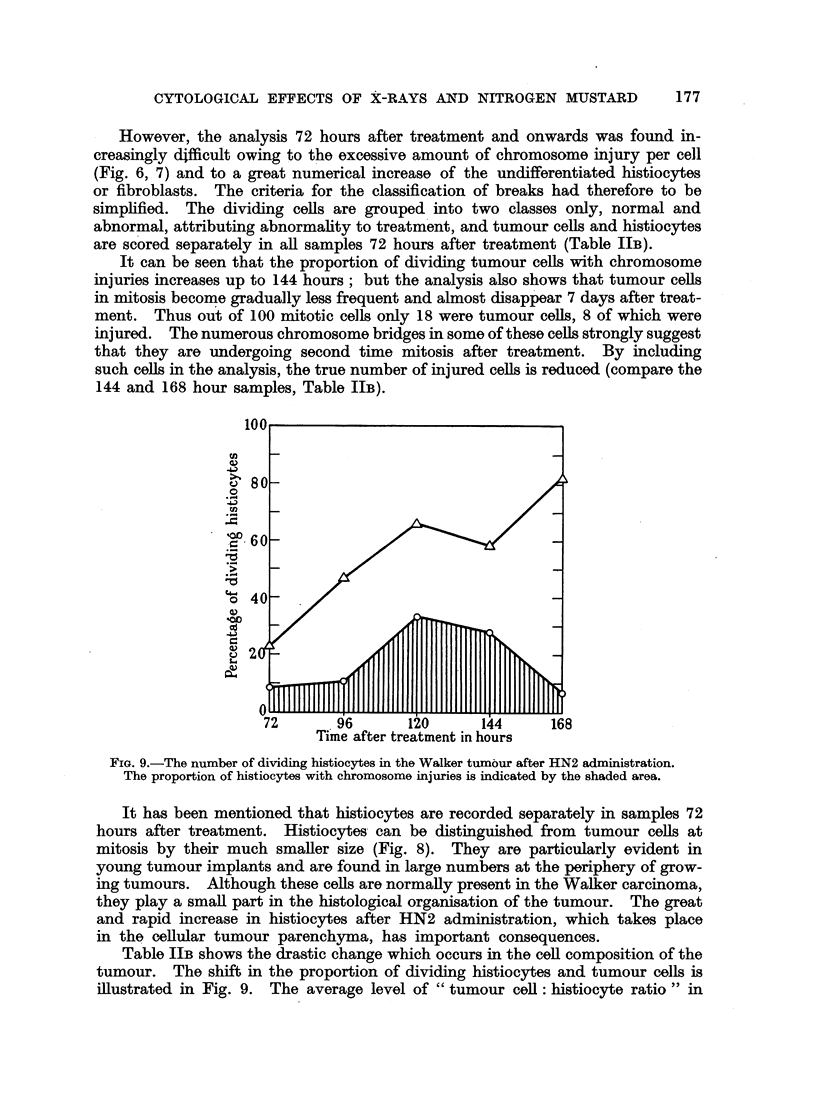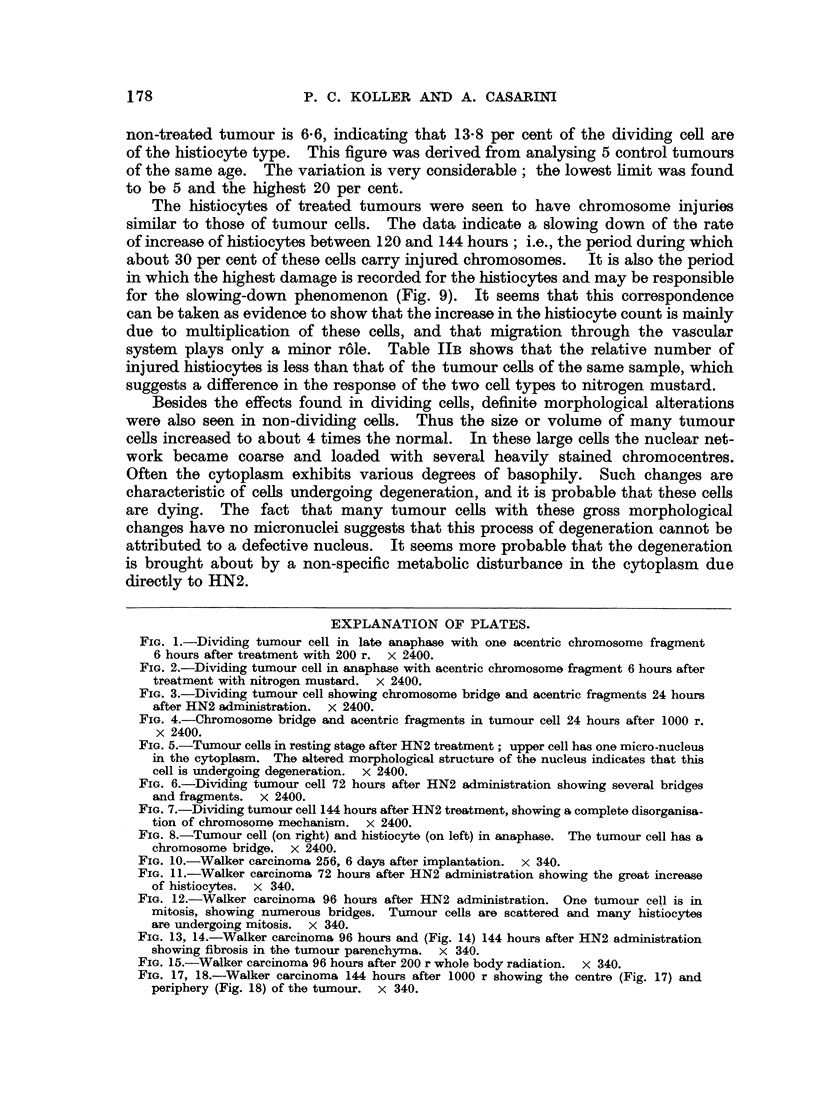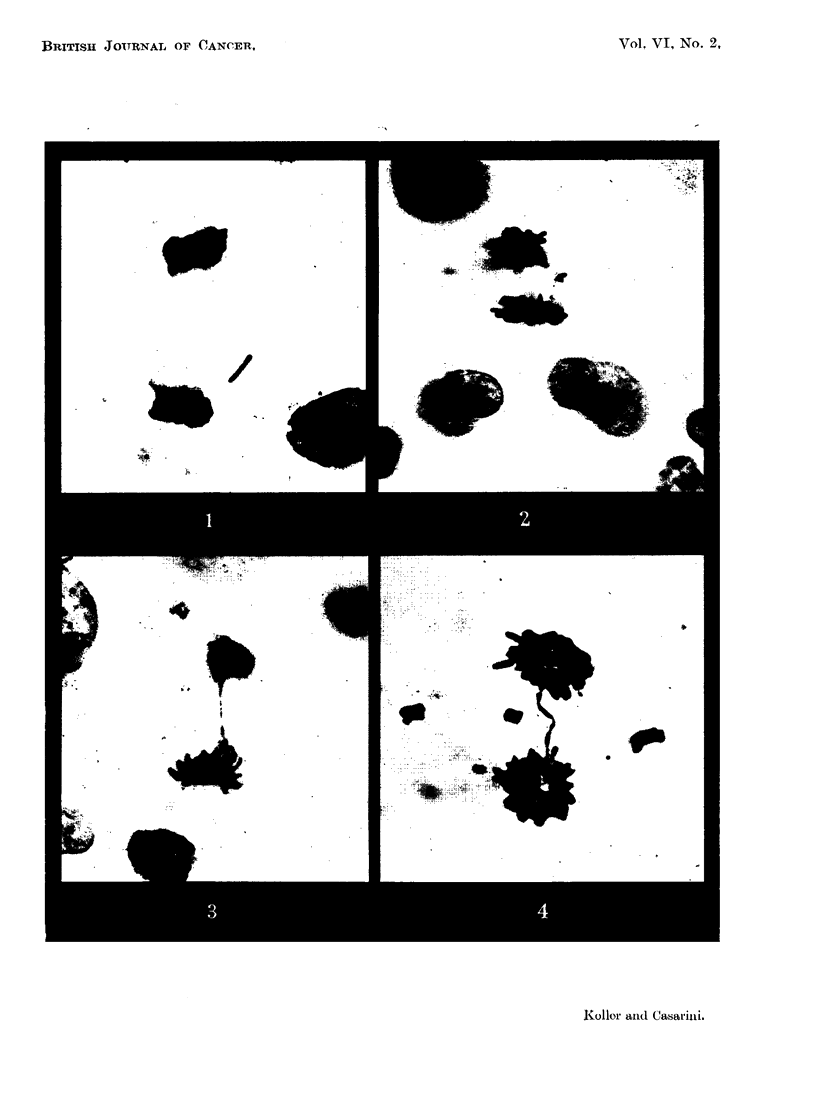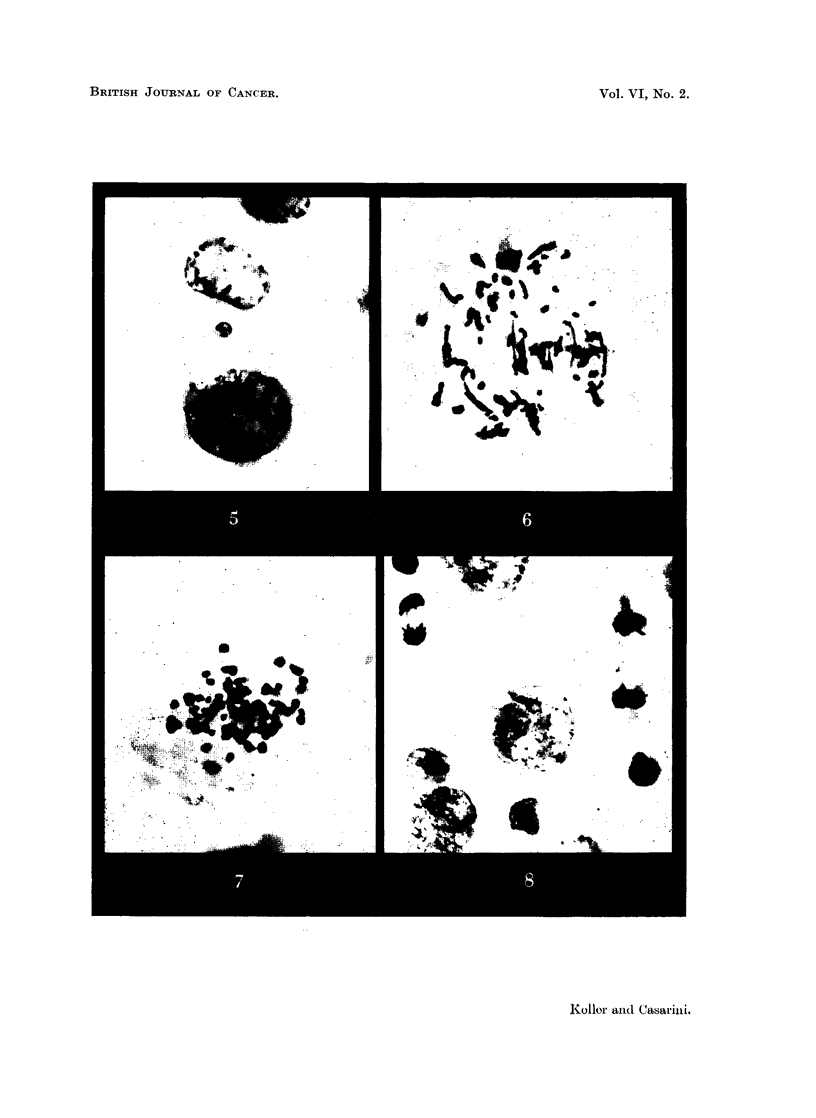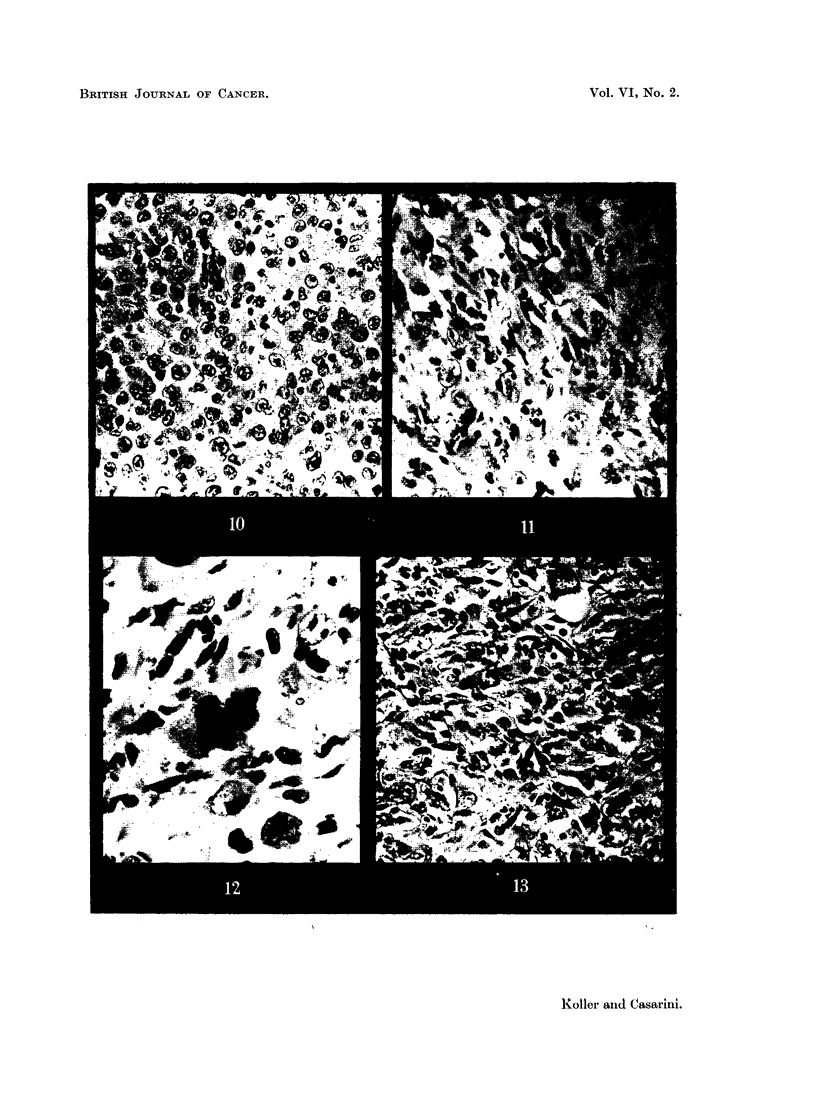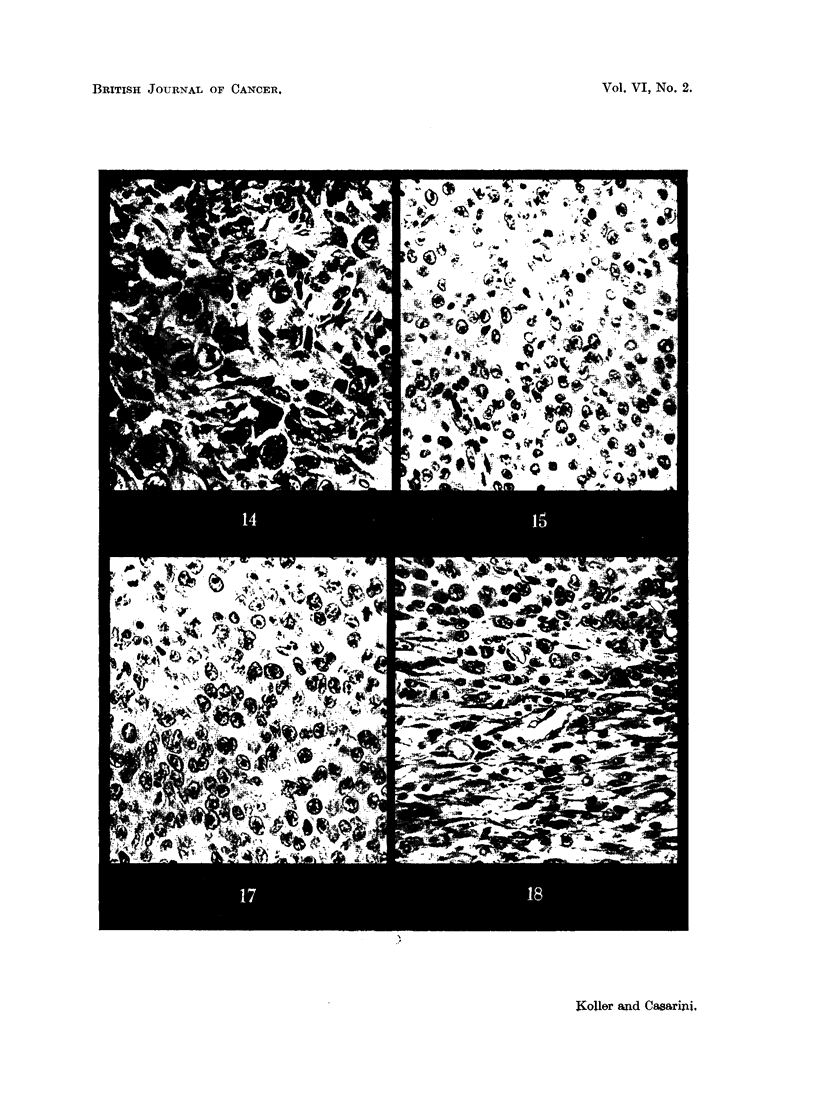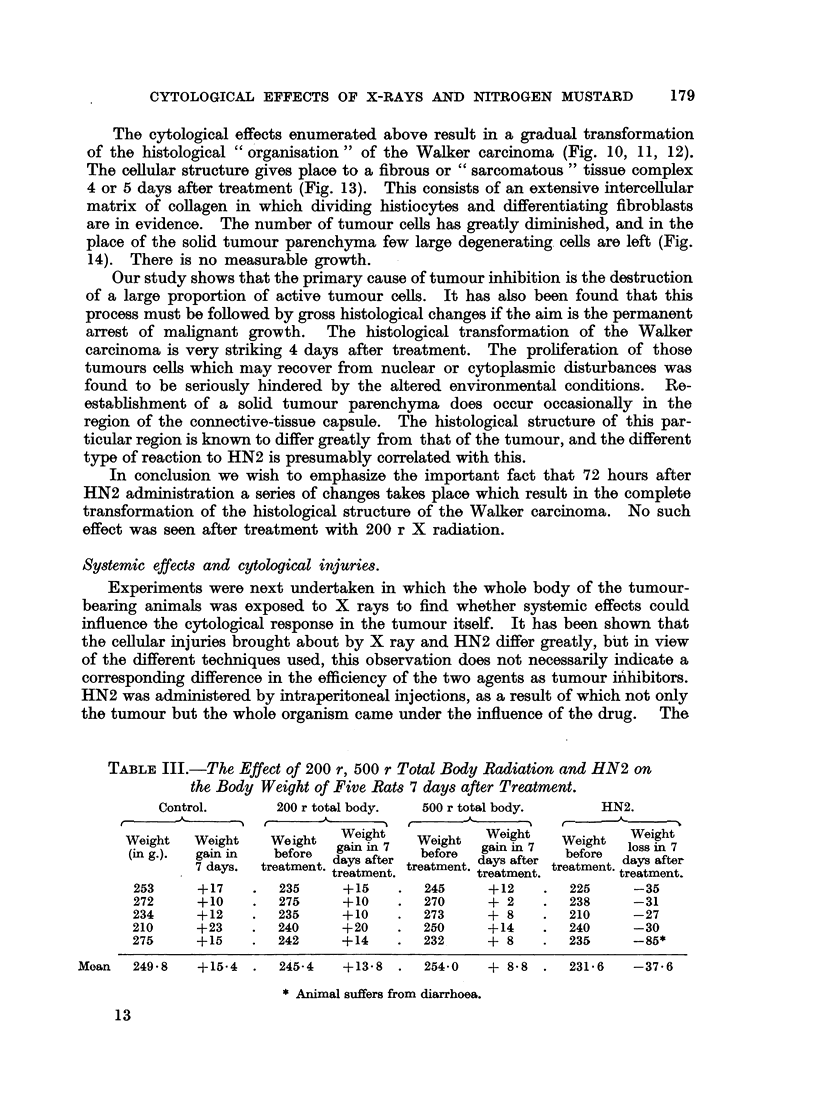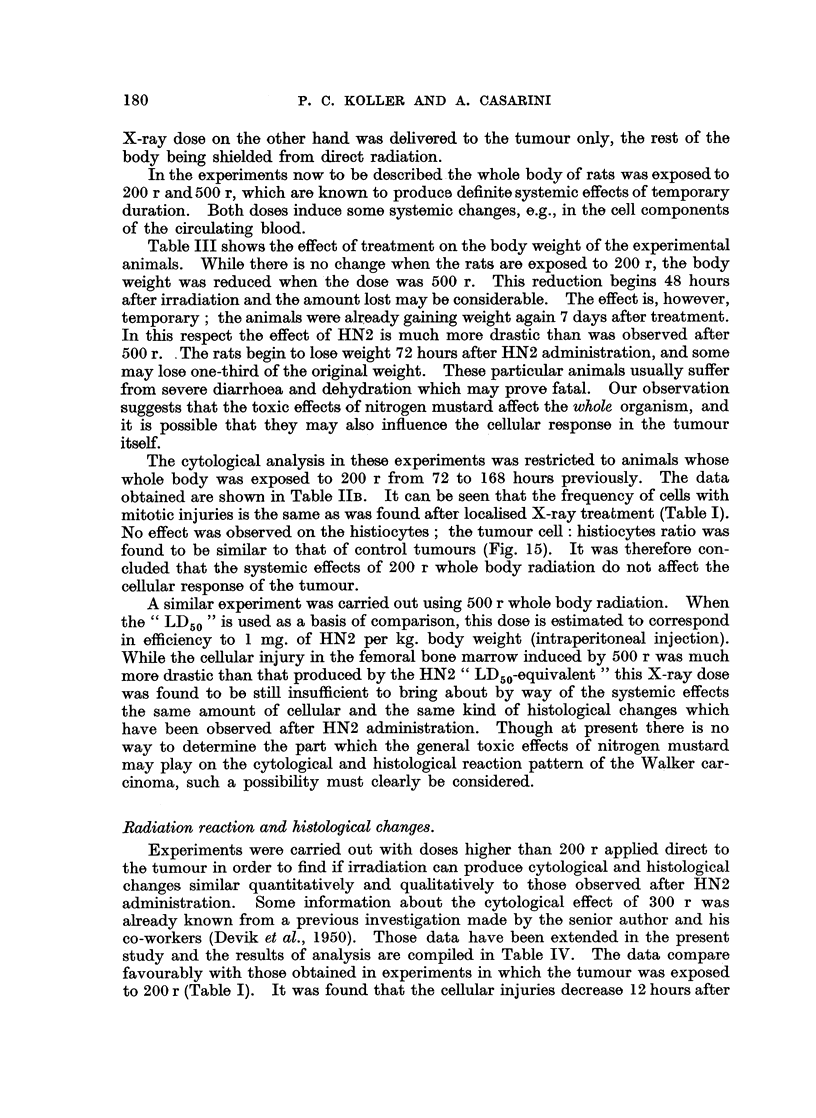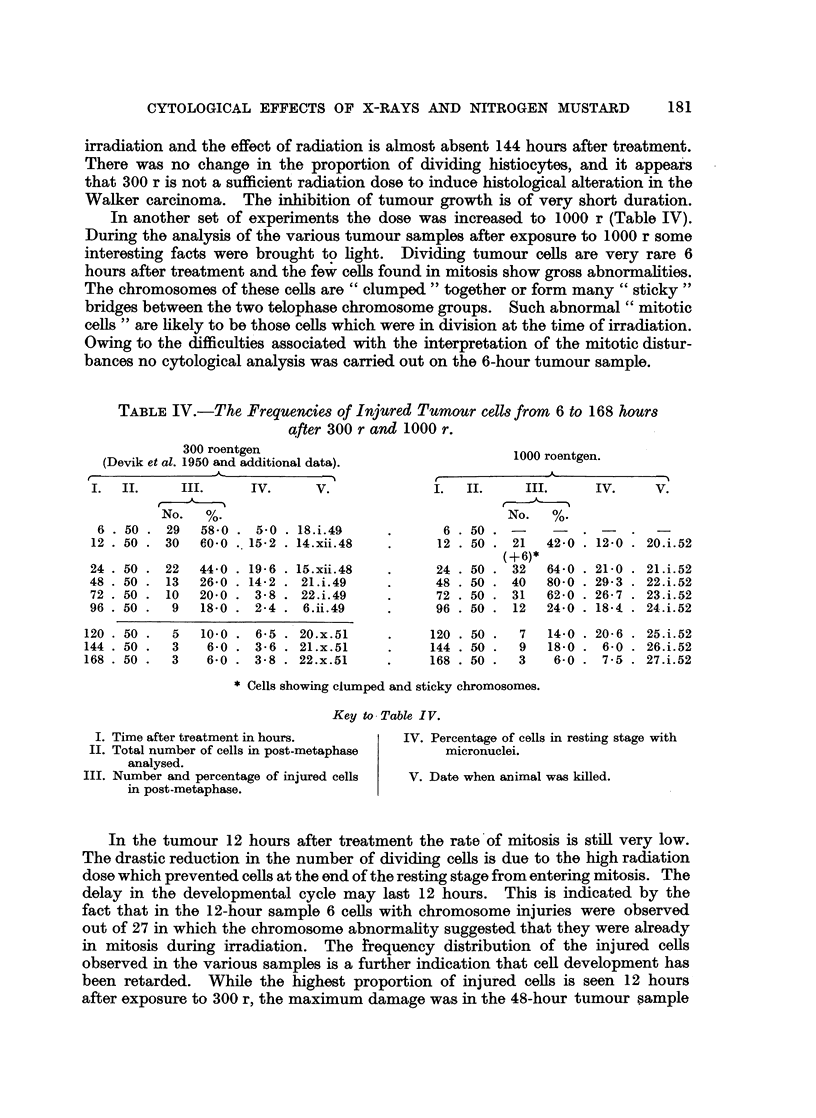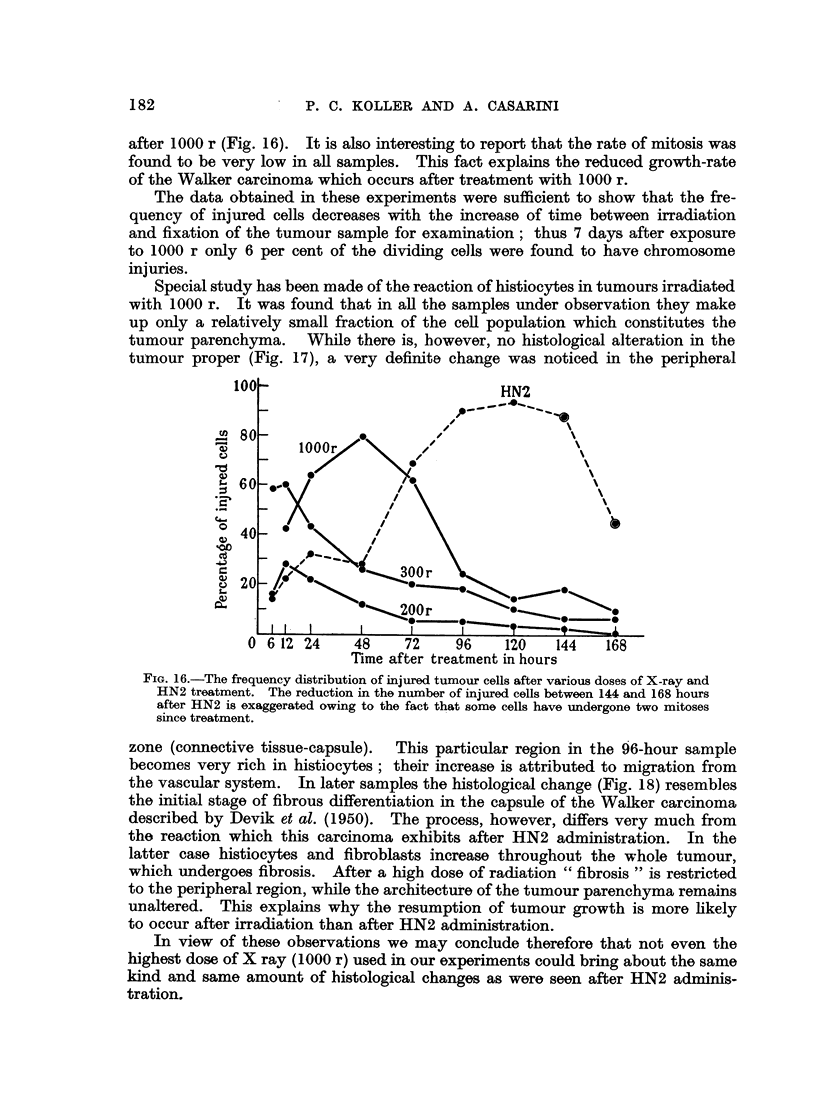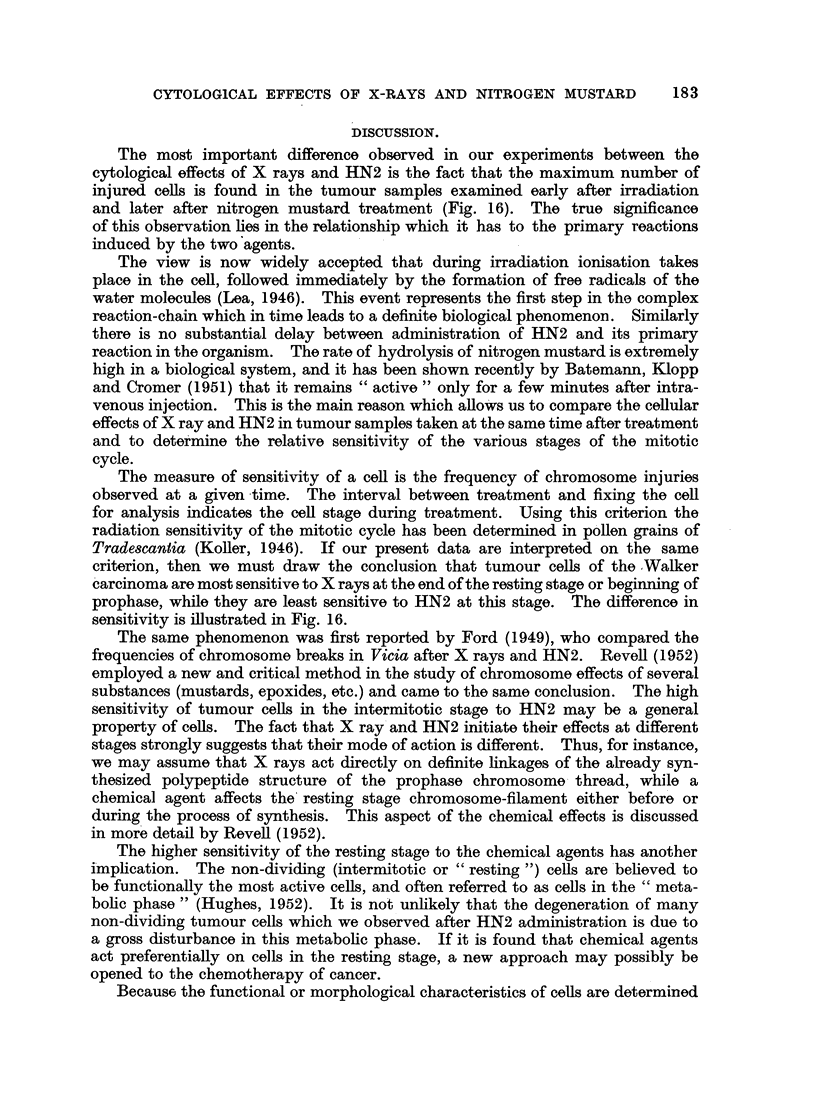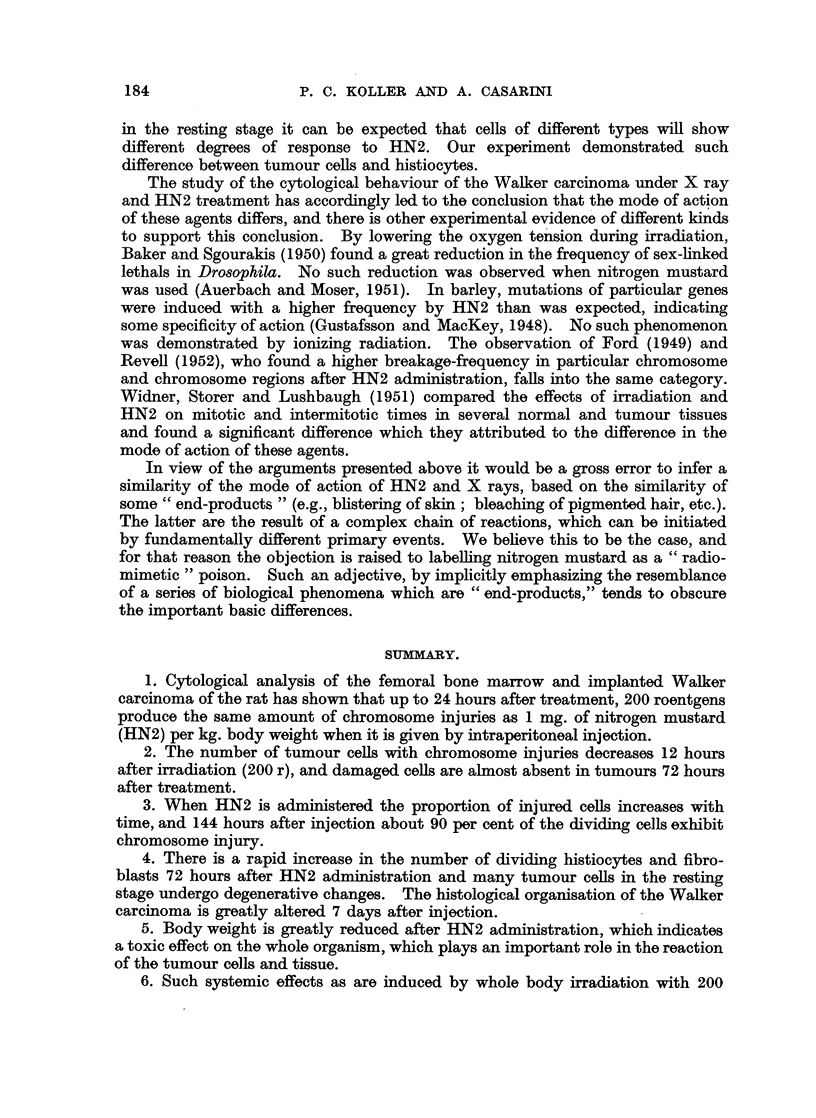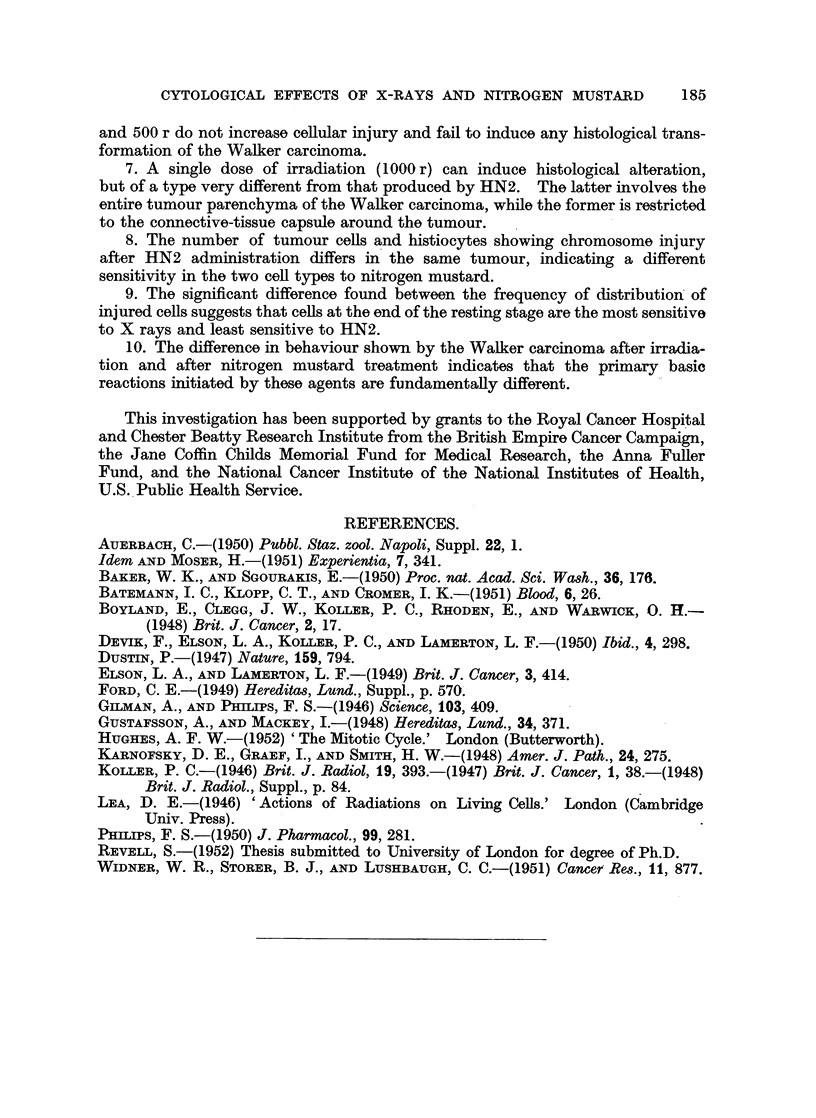# Comparison of Cytological Effects Induced by X Rays and Nitrogen Mustard

**DOI:** 10.1038/bjc.1952.20

**Published:** 1952-06

**Authors:** P. C. Koller, A. Casarini

## Abstract

**Images:**


### COMPARISON OF CYTOLOGICAL EFFECTS INDUCED BY X RAYS AND NITROGEN MUSTARD.

P. C. KOLLER AND A. CASARINI.

*From the Chester Beatty Research Institute, The Royal Cancer Hospital, London, S.W.3.*

Received for publication May 2, 1952.

THE possibility that the " mustards " might be of biological interest was first suspected during the last war. At that time research into their pharmacological and toxicological properties showed that certain of their effects were strikingly similar to those of ionizing radiation (Gilman and Philips, 1946 ; Karnofsky, Graef and Smith, 1948 ; Philips, 1950). This led to further investigations of their action as mutagenic agents and as growth inhibitors and their importance in these connections quickly became apparent. On account of the great similarity shown by the effects of mustards to those of irradiation the mustards were labelled as " radiomimetic poisons " (Dustin, 1947).

So far as their mutagenic effects are concerned, these compounds have been found to be highly active. Like X rays they are able to produce genetic changes at dose levels far below those at which any toxicological change can be demonstrated by present biochemical techniques. However, the types of genetic change which they induce are in some respects different from those caused by X rays, and evidence is now accumulating which strongly suggests that the mode of action of the two agents is also very different (Auerbach, 1950).

During the present investigation the cytological effects induced by X rays and one of the mustards (methyl-bis-(β chloroethyl)amine or HN2) in tumour cells of the transplantable Walker carcinoma 256 have been analysed and compared. These effects, consisting primarily of chromosome fragmentation, were found by Koller (1948) to underlie inhibition of normal and malignant growth in tissues. Our aim was to throw some light on the basic biological reaction which results in temporary or permanent inhibition of growth, and to ascertain if possible in what respects the mode of action of X rays and nitrogen mustard differ in producing their inhibition.

### EXPERIMENTAL TECHNIQUE.

Walker carcinoma 256 was employed for all experiments. It was grafted subcutaneously into white albino rats fed *ad libitum* on routine mixed diet containing about 15 per cent protein. Animals of both sexes were used weighing from 150 to 230 g. Tumours were selected for treatment on the 6th or 7th day after implantation.

The standard dose level adapted as the basis for comparison of chemical and X-ray effects was 1 mg. of HN2 per kg. body weight. In preliminary experiments the chromosome effects of this dose in femoral bone marrow and tumour were compared with the effects of 100, 200, 400 and 500 r whole body irradiation. These analyses showed that 200 r might be considered to be an " equivalent " dose, i.e., it produced in tumour and bone marrow the same amount and the same type of cellular injuries up to 24 hours after administration (Koller, unpublished). The previously reported X-ray " equivalent " dose based on the 50 per cent lethal dose (LD 50) is about 400–500 r.

In the first set of experiments irradiation with 200 r was applied direct to the tumour. The technical conditions were as follows : 140 kV 0·1 mm. Cu filter, focal skin distance of applicator 13 cm., dosage rate 140 r/min. (Elson and Lamerton, 1949). During the investigation it was found that beside the cellular effects HN2 also produced significant histological changes. It was suspected that systemic toxic effects of HN2 might be responsible for this additional effect. In order to study the influence of systemic effects of X rays on cellular reaction in another set of experiments 200 r (and 500 r) was given as whole body irradiation under the following conditions : 220 kV half value layer 1·5 mm. Cu, focal skin distance 90 cm., dosage rate 7·5 r/min.

When it was found that the toxic effects of 200 r whole body irradiation did not increase cellular reaction, tumours were exposed to 300 and 1000 r in order to find if doses higher than 200 r can produce histological changes similar to those observed after HN2 administration. These experiments in which various doses and methods of irradiation are used have been undertaken to demonstrate the widest possible spectrum of the biological reactions which can be brought about by X rays in the Walker carcinoma.

Nitrogen mustard was administered by single intraperitoneal injection using freshly prepared aqueous solution of 0·02 per cent of the commercial compound methyl-bis-(β chloroethyl) amine hydrochloride (Boots & Co.). The dose was 1 mg. per kg. body weight throughout the experiments.

For cytological analysis small pieces of tumours were fixed in acetic alcohol mixture (1 : 3), and squash preparations were made and stained with Feulgen's basic fuchsin. For histological study fragments were fixed in Bouin's solution, embedded in paraffin wax, and sections cut at 5μ were stained with haematoxylin and orange-G.

*Cytological effects : method of analysis.*

The method used for estimating cellular injuries is the same as was employed by Devik, Elson, Koller and Lamerton (1950). Cells in post-metaphase stages were selected. At this stage the " acentric " fragments can be seen, scattered between the two daughter chromosome groups (Fig. 1, 2). " Dicentrics " are seen as chromosome bridges at this stage and these also were recorded (Fig. 3, 4). The " micronuclei " of resting cells are a further criteria of mitotic injury ; they represent chromosome fragments left in the cytoplasm at the completion of mitosis (Fig. 5). None of these abnormalities occur except as an extreme rarity in control tumours ; in two control experiments we found only 3 and 5 per cent of cells with any mitotic disturbance and these were mostly cases of stickiness of chromosomes at anaphase. These have, of course, an origin quite different from that of true bridges, brought about by dicentric chromosomes or chromatids (Koller, 1947), and they could be distinguished and classified accordingly.

It should be mentioned that the tumour-bearing animals were unrelated and were treated and killed at varying times. Their genetic heterogeneity is reflected in the variation shown by the quantitative data. The importance of some biological variables (age, sex, diet, etc.) has already been discussed ; Devik and co-workers (1950) demonstrated by analysis that the data, obtained by the same method as used in the present study, do in fact provide a valid basis for comparison. But as will be seen, the effects observed 72 hours after treatment by X ray and HN2 were in any case qualitatively so different that they could not be compared on a quantitative basis.

*X-ray effects : localised treatment* (200 *r*).

The tumours only were irradiated and cytological effects were analysed at 6, 12, 24, 48, 72, 96, 120, 144 and 168 hours after treatment. The data obtained are presented in Table I. It can be seen that the number of cells with chromosome injuries is highest in the tumour sample which was taken 12 hours after treatment.

TABLE I.—*The Various Cell Injuries Induced in the Walker Carcinoma after* 200*r*.

| I. | II. | III. | IV. | | V. | | | | | | VI. | VII. | VIII. | IX. |
|---|---|---|---|---|---|---|---|---|---|---|---|---|---|---|
| | | | No. | %. | 0. | 1. | 2. | 3. | 4. | 5+. | | | | |
| 200 r (tumour) | 6 | 50 | 8 | 16·0 | 44 | 3 | 3 | 0 | 0 | 0 | 5 | 0·18 | 1·6 | 5.xii.50 |
| | 12 | 50 | 14 | 28·0 | 39 | 7 | 4 | 0 | 0 | 0 | 5 | 0·30 | 8·4 | 5.xii.50 |
| | 24 | 50 | 11 | 22·0 | 41 | 7 | 1 | 1 | 0 | 0 | 3 | 0·24 | 7·2 | 5.xii.50 |
| | 48 | 50 | 16 | 12·0 | 47 | 2 | 0 | 1 | 0 | 0 | 3 | 0·10 | 2·6 | 7.xii.50 |
| | 72 | 100 | 5 | 5·0 | 97 | 2 | 1 | 0 | 0 | 0 | 2 | 0·04 | 3·0 | 18.v.51 |
| | 96 | 100 | 5 | 5·0 | 97 | 2 | 1 | 0 | 0 | 0 | 2 | 0·04 | 1·1 | 19.v.51 |
| | 120 | 100 | 3 | 3·0 | 98 | 2 | 0 | 0 | 0 | 0 | 1 | 0·02 | 0·5 | 20.v.51 |
| | 144 | 100 | 2 | 2·0 | 98 | 1 | 1 | 0 | 0 | 0 | 0 | 0·03 | 0·4 | 21.v.51 |
| | 168 | 100 | (2)* | 0·0 | 0 | 0 | 0 | 0 | 0 | 0 | 0 | 0 | 0·5 | 22.v.51 |

* Sticky chromosome bridges, probably not due to X ray.

*Key to Table I.*

I. X-ray dose in roentgens or HN2 dose in mg/kg.
II. Time after treatment in hours.
III. Total number of cells in post-metaphase analysed.
IV. Number and percentage of injured cells in post-metaphase.
V. Number of cells with 0– 5+ fragments.
VI. Number of cells with bridges.
VII. Number of fragments per cell.
VIII. Percentage of cells in resting stage with micronuclei.
IX. Date when the animal was killed.

This is also the sample in which the number of chromosome fragments per cell is at a maximum. The damage is less 24 hours after irradiation, and it diminishes further in the 48 and 72-hour samples. In tumours 4 to 6 days after treatment the number of dividing tumour cells with abnormalities is very small. Our observations indicate that 200 r is insufficient to inhibit the growth of the Walker carcinoma ; the growth rate of treated tumours 6 days after irradiation was found to be the same as that of the controls. This finding is in close agreement with that of Devik and co-workers (1950).

*HN2-induced cytological effects.*

It has already been reported by the senior author that a dose of 1 mg. per kg. body weight of HN2 produces chromosome fragmentation in cells of the Walker carcinoma (Boyland, Clegg, Koller, Rhoden and Warwick, 1948). In the present experiments the same dose was given, and it was intended to carry out an analysis similar to that of the X-rayed tumours. The type and amount of injury was successfully analysed in the 6, 12, 24 and 48-hour tumour samples (Table IIA), and the comparison of these data with those from X-rayed tumours is fairly straightforward. The total number of tumour cells showing chromosome injuries

TABLE IIA.—*The Various Cell Injuries Induced in the Walker Carcinoma from 6 to 48 hours after HN2 Administration.*

| I. | II. | III. | IV. | | V. | | | | | | VI. | VII. | VIII. | IX. |
|---|---|---|---|---|---|---|---|---|---|---|---|---|---|---|
| | | | No. | %. | 0. | 1. | 2. | 3. | 4. | 5+. | | | | |
| HN2 1 mg. per kg. body weight | 6 | 50 | 7 | 14·0 | 47 | 2 | 1 | 0 | 0 | 0 | 4 | 0·08 | 0·8 | 5.xii.50 |
| | 12 | 50 | 11 | 22·0 | 42 | 4 | 3 | 0 | 0 | 1 | 4 | 0·40 | 0·2 | 5.xii.50 |
| | 24 | 50 | 16 | 32·0 | 34 | 2 | 2 | 0 | 2 | 10 | 9 | 2·12 | 2·2 | 6.xii.50 |
| | 48 | 50 | 14 | 28·0 | 37 | 2 | 2 | 0 | 2 | 7 | 10 | 2·30 | 14·8 | 7.xii.50 |

*Key to Table IIA.*—Same as that of Table I.

TABLE IIB.—*The Frequency of Injured Tumour Cells and Histiocytes from 72 to 168 hours after HN2 and 200 r Whole Body Irradiation.*

| | | | Tumour cells. | | | | Histiocytes. | | | | |
|---|---|---|---|---|---|---|---|---|---|---|---|
| I. | II. | III. | IV. | V. | | VI. | VII. | VIII. | | IX.* | X. |
| | | | | No. | %. | | | No. | %. | | |
| HN2 1 mg. per kg. body weight | 72 | 100 | 77 | 42 | 54·7 | 15·9 | 23 | 2 | 8·7 | 3·3 | 24.v.51 |
| | 96 | 100 | 53 | 48 | 90·5 | 19·9 | 47 | 5 | 10·6 | 1·1 | 25.v.51 |
| | 120 | 100 | 34 | 32 | 94·0 | 24·1 | 66 | 20 | 30·3 | 0·5 | 14.ix.51 |
| | 144 | 100 | 42 | 37 | 88·0 | 23·0 | 58 | 16 | 27·4 | 0·7 | 15.ix.51 |
| | 168 | 100 | 18 | 8 | 44·4 | 31·2 | 82 | 8 | 9·7 | 0·2 | 16.ix.51 |
| 200 r (Whole body) | 72 | 100 | 88 | 4 | 4·5 | 8·9 | 12 | 2 | (16·6) | 7·3 | 1.vi.51 |
| | 96 | 100 | 93 | 8 | 8·6 | 2·9 | 7 | 0 | — | 13·2 | 2.vi.51 |
| | 120 | 100 | 83 | 3 | 3·6 | 1·2 | 17 | 1 | (5·8) | 12·4 | 3.vi.51 |
| | 144 | 100 | 85 | 7 | 8·4 | 1·2 | 15 | 2 | (13·3) | 5·6 | 4.vi.51 |
| | 168 | 100 | 90 | 2 | 2·2 | 1·1 | 10 | 0 | — | 9·0 | 5.vi.51 |

* $\frac{\text{Tumour cell}}{\text{Histiocyte}}$ ratio in control tumour is 6·6.

*Key to Table IIB.*

I. HN2 dose in mg/kg. and 200 r (whole body) treatment.
II. Time after treatment in hours.
III. Total number of cells in post-metaphase stages analysed.
IV. Total number of tumour cells in post-metaphase stages.
V. Number and percentage of injured cells in post-metaphase stages.
VI. Percentage of cells in resting stage with micronuclei.
VII. Total number of histiocytes in post-metaphase stages.
VIII. Number and percentage of injured cells in post-metaphase stages.
IX. Ratio of dividing tumour cell and histiocytes $\frac{(T)}{H}$.
X. Date when the animal was killed.

up to 24 hours after treatment is 34 out of 150 cells analysed. It is the same as was found after irradiation with 200 r (33 out of 150). It can be seen, however, that breaks appear somewhat more slowly after HN2 than after X-ray treatment. The number of cells with HN2-induced injuries increases gradually from 6 to 48 hours; after that period their number increases rapidly. This is the first significant difference which appears between the effects of HN2 and X rays.

However, the analysis 72 hours after treatment and onwards was found increasingly difficult owing to the excessive amount of chromosome injury per cell (Fig. 6, 7) and to a great numerical increase of the undifferentiated histiocytes or fibroblasts. The criteria for the classification of breaks had therefore to be simplified. The dividing cells are grouped into two classes only, normal and abnormal, attributing abnormality to treatment, and tumour cells and histiocytes are scored separately in all samples 72 hours after treatment (Table IIB).

It can be seen that the proportion of dividing tumour cells with chromosome injuries increases up to 144 hours ; but the analysis also shows that tumour cells in mitosis become gradually less frequent and almost disappear 7 days after treatment. Thus out of 100 mitotic cells only 18 were tumour cells, 8 of which were injured. The numerous chromosome bridges in some of these cells strongly suggest that they are undergoing second time mitosis after treatment. By including such cells in the analysis, the true number of injured cells is reduced (compare the 144 and 168 hour samples, Table IIB).

FIG. 9.—The number of dividing histiocytes in the Walker tumour after HN2 administration. The proportion of histiocytes with chromosome injuries is indicated by the shaded area.

It has been mentioned that histiocytes are recorded separately in samples 72 hours after treatment. Histiocytes can be distinguished from tumour cells at mitosis by their much smaller size (Fig. 8). They are particularly evident in young tumour implants and are found in large numbers at the periphery of growing tumours. Although these cells are normally present in the Walker carcinoma, they play a small part in the histological organisation of the tumour. The great and rapid increase in histiocytes after HN2 administration, which takes place in the cellular tumour parenchyma, has important consequences.

Table IIB shows the drastic change which occurs in the cell composition of the tumour. The shift in the proportion of dividing histiocytes and tumour cells is illustrated in Fig. 9. The average level of " tumour cell : histiocyte ratio " in non-treated tumour is 6·6, indicating that 13·8 per cent of the dividing cell are of the histiocyte type. This figure was derived from analysing 5 control tumours of the same age. The variation is very considerable ; the lowest limit was found to be 5 and the highest 20 per cent.

The histiocytes of treated tumours were seen to have chromosome injuries similar to those of tumour cells. The data indicate a slowing down of the rate of increase of histiocytes between 120 and 144 hours ; i.e., the period during which about 30 per cent of these cells carry injured chromosomes. It is also the period in which the highest damage is recorded for the histiocytes and may be responsible for the slowing-down phenomenon (Fig. 9). It seems that this correspondence can be taken as evidence to show that the increase in the histiocyte count is mainly due to multiplication of these cells, and that migration through the vascular system plays only a minor rôle. Table IIB shows that the relative number of injured histiocytes is less than that of the tumour cells of the same sample, which suggests a difference in the response of the two cell types to nitrogen mustard.

Besides the effects found in dividing cells, definite morphological alterations were also seen in non-dividing cells. Thus the size or volume of many tumour cells increased to about 4 times the normal. In these large cells the nuclear network became coarse and loaded with several heavily stained chromocentres. Often the cytoplasm exhibits various degrees of basophily. Such changes are characteristic of cells undergoing degeneration, and it is probable that these cells are dying. The fact that many tumour cells with these gross morphological changes have no micronuclei suggests that this process of degeneration cannot be attributed to a defective nucleus. It seems more probable that the degeneration is brought about by a non-specific metabolic disturbance in the cytoplasm due directly to HN2.

### EXPLANATION OF PLATES.

FIG. 1.—Dividing tumour cell in late anaphase with one acentric chromosome fragment 6 hours after treatment with 200 r. × 2400.

FIG. 2.—Dividing tumour cell in anaphase with acentric chromosome fragment 6 hours after treatment with nitrogen mustard. × 2400.

FIG. 3.—Dividing tumour cell showing chromosome bridge and acentric fragments 24 hours after HN2 administration. × 2400.

FIG. 4.—Chromosome bridge and acentric fragments in tumour cell 24 hours after 1000 r. × 2400.

FIG. 5.—Tumour cells in resting stage after HN2 treatment ; upper cell has one micro-nucleus in the cytoplasm. The altered morphological structure of the nucleus indicates that this cell is undergoing degeneration. × 2400.

FIG. 6.—Dividing tumour cell 72 hours after HN2 administration showing several bridges and fragments. × 2400.

FIG. 7.—Dividing tumour cell 144 hours after HN2 treatment, showing a complete disorganisation of chromosome mechanism. × 2400.

FIG. 8.—Tumour cell (on right) and histiocyte (on left) in anaphase. The tumour cell has a chromosome bridge. × 2400.

FIG. 10.—Walker carcinoma 256, 6 days after implantation. × 340.

FIG. 11.—Walker carcinoma 72 hours after HN2 administration showing the great increase of histiocytes. × 340.

FIG. 12.—Walker carcinoma 96 hours after HN2 administration. One tumour cell is in mitosis, showing numerous bridges. Tumour cells are scattered and many histiocytes are undergoing mitosis. × 340.

FIG. 13, 14.—Walker carcinoma 96 hours and (Fig. 14) 144 hours after HN2 administration showing fibrosis in the tumour parenchyma. × 340.

FIG. 15.—Walker carcinoma 96 hours after 200 r whole body radiation. × 340.

FIG. 17, 18.—Walker carcinoma 144 hours after 1000 r showing the centre (Fig. 17) and periphery (Fig. 18) of the tumour. × 340.

Koller and Casarini.

Koller and Casarini.

10

11

12

13

14

15

17

18

The cytological effects enumerated above result in a gradual transformation of the histological " organisation " of the Walker carcinoma (Fig. 10, 11, 12). The cellular structure gives place to a fibrous or " sarcomatous " tissue complex 4 or 5 days after treatment (Fig. 13). This consists of an extensive intercellular matrix of collagen in which dividing histiocytes and differentiating fibroblasts are in evidence. The number of tumour cells has greatly diminished, and in the place of the solid tumour parenchyma few large degenerating cells are left (Fig. 14). There is no measurable growth.

Our study shows that the primary cause of tumour inhibition is the destruction of a large proportion of active tumour cells. It has also been found that this process must be followed by gross histological changes if the aim is the permanent arrest of malignant growth. The histological transformation of the Walker carcinoma is very striking 4 days after treatment. The proliferation of those tumours cells which may recover from nuclear or cytoplasmic disturbances was found to be seriously hindered by the altered environmental conditions. Re-establishment of a solid tumour parenchyma does occur occasionally in the region of the connective-tissue capsule. The histological structure of this particular region is known to differ greatly from that of the tumour, and the different type of reaction to HN2 is presumably correlated with this.

In conclusion we wish to emphasize the important fact that 72 hours after HN2 administration a series of changes takes place which result in the complete transformation of the histological structure of the Walker carcinoma. No such effect was seen after treatment with 200 r X radiation.

*Systemic effects and cytological injuries.*

Experiments were next undertaken in which the whole body of the tumour-bearing animals was exposed to X rays to find whether systemic effects could influence the cytological response in the tumour itself. It has been shown that the cellular injuries brought about by X ray and HN2 differ greatly, but in view of the different techniques used, this observation does not necessarily indicate a corresponding difference in the efficiency of the two agents as tumour inhibitors. HN2 was administered by intraperitoneal injections, as a result of which not only the tumour but the whole organism came under the influence of the drug. The

TABLE III.—*The Effect of* 200 *r*, 500 *r Total Body Radiation and HN*2 *on the Body Weight of Five Rats* 7 *days after Treatment.*

| | Control. | | 200 r total body. | | 500 r total body. | | HN2. | |
|---|---|---|---|---|---|---|---|---|
| | Weight (in g.). | Weight gain in 7 days. | Weight before treatment. | Weight gain in 7 days after treatment. | Weight before treatment. | Weight gain in 7 days after treatment. | Weight before treatment. | Weight loss in 7 days after treatment. |
| | 253 | +17 | 235 | +15 | 245 | +12 | 225 | −35 |
| | 272 | +10 | 275 | +10 | 270 | + 2 | 238 | −31 |
| | 234 | +12 | 235 | +10 | 273 | + 8 | 210 | −27 |
| | 210 | +23 | 240 | +20 | 250 | +14 | 240 | −30 |
| | 275 | +15 | 242 | +14 | 232 | + 8 | 235 | −85* |
| Mean | 249·8 | +15·4 | 245·4 | +13·8 | 254·0 | + 8·8 | 231·6 | −37·6 |

* Animal suffers from diarrhoea.

X-ray dose on the other hand was delivered to the tumour only, the rest of the body being shielded from direct radiation.

In the experiments now to be described the whole body of rats was exposed to 200 r and 500 r, which are known to produce definite systemic effects of temporary duration. Both doses induce some systemic changes, e.g., in the cell components of the circulating blood.

Table III shows the effect of treatment on the body weight of the experimental animals. While there is no change when the rats are exposed to 200 r, the body weight was reduced when the dose was 500 r. This reduction begins 48 hours after irradiation and the amount lost may be considerable. The effect is, however, temporary ; the animals were already gaining weight again 7 days after treatment. In this respect the effect of HN2 is much more drastic than was observed after 500 r. The rats begin to lose weight 72 hours after HN2 administration, and some may lose one-third of the original weight. These particular animals usually suffer from severe diarrhoea and dehydration which may prove fatal. Our observation suggests that the toxic effects of nitrogen mustard affect the *whole* organism, and it is possible that they may also influence the cellular response in the tumour itself.

The cytological analysis in these experiments was restricted to animals whose whole body was exposed to 200 r from 72 to 168 hours previously. The data obtained are shown in Table IIB. It can be seen that the frequency of cells with mitotic injuries is the same as was found after localised X-ray treatment (Table I). No effect was observed on the histiocytes ; the tumour cell : histiocytes ratio was found to be similar to that of control tumours (Fig. 15). It was therefore concluded that the systemic effects of 200 r whole body radiation do not affect the cellular response of the tumour.

A similar experiment was carried out using 500 r whole body radiation. When the " $LD_{50}$ " is used as a basis of comparison, this dose is estimated to correspond in efficiency to 1 mg. of HN2 per kg. body weight (intraperitoneal injection). While the cellular injury in the femoral bone marrow induced by 500 r was much more drastic than that produced by the HN2 " $LD_{50}$-equivalent " this X-ray dose was found to be still insufficient to bring about by way of the systemic effects the same amount of cellular and the same kind of histological changes which have been observed after HN2 administration. Though at present there is no way to determine the part which the general toxic effects of nitrogen mustard may play on the cytological and histological reaction pattern of the Walker carcinoma, such a possibility must clearly be considered.

*Radiation reaction and histological changes.*

Experiments were carried out with doses higher than 200 r applied direct to the tumour in order to find if irradiation can produce cytological and histological changes similar quantitatively and qualitatively to those observed after HN2 administration. Some information about the cytological effect of 300 r was already known from a previous investigation made by the senior author and his co-workers (Devik *et al.*, 1950). Those data have been extended in the present study and the results of analysis are compiled in Table IV. The data compare favourably with those obtained in experiments in which the tumour was exposed to 200 r (Table I). It was found that the cellular injuries decrease 12 hours after irradiation and the effect of radiation is almost absent 144 hours after treatment. There was no change in the proportion of dividing histiocytes, and it appears that 300 r is not a sufficient radiation dose to induce histological alteration in the Walker carcinoma. The inhibition of tumour growth is of very short duration.

In another set of experiments the dose was increased to 1000 r (Table IV). During the analysis of the various tumour samples after exposure to 1000 r some interesting facts were brought to light. Dividing tumour cells are very rare 6 hours after treatment and the few cells found in mitosis show gross abnormalities. The chromosomes of these cells are " clumped " together or form many " sticky " bridges between the two telophase chromosome groups. Such abnormal " mitotic cells " are likely to be those cells which were in division at the time of irradiation. Owing to the difficulties associated with the interpretation of the mitotic disturbances no cytological analysis was carried out on the 6-hour tumour sample.

TABLE IV.—*The Frequencies of Injured Tumour cells from* 6 *to* 168 *hours after* 300 *r and* 1000 *r.*

| 300 roentgen (Devik *et al.* 1950 and additional data). | | | | | | | 1000 roentgen. | | | | | |
|---|---|---|---|---|---|---|---|---|---|---|---|---|
| I. | II. | III. | | IV. | V. | | I. | II. | III. | | IV. | V. |
| | | No. | %. | | | | | | No. | %. | | |
| 6 | 50 | 29 | 58·0 | 5·0 | 18.i.49 | | 6 | 50 | — | — | — | — |
| 12 | 50 | 30 | 60·0 | 15·2 | 14.xii.48 | | 12 | 50 | 21 (+6)* | 42·0 | 12·0 | 20.i.52 |
| 24 | 50 | 22 | 44·0 | 19·6 | 15.xii.48 | | 24 | 50 | 32 | 64·0 | 21·0 | 21.i.52 |
| 48 | 50 | 13 | 26·0 | 14·2 | 21.i.49 | | 48 | 50 | 40 | 80·0 | 29·3 | 22.i.52 |
| 72 | 50 | 10 | 20·0 | 3·8 | 22.i.49 | | 72 | 50 | 31 | 62·0 | 26·7 | 23.i.52 |
| 96 | 50 | 9 | 18·0 | 2·4 | 6.ii.49 | | 96 | 50 | 12 | 24·0 | 18·4 | 24.i.52 |
| 120 | 50 | 5 | 10·0 | 6·5 | 20.x.51 | | 120 | 50 | 7 | 14·0 | 20·6 | 25.i.52 |
| 144 | 50 | 3 | 6·0 | 3·6 | 21.x.51 | | 144 | 50 | 9 | 18·0 | 6·0 | 26.i.52 |
| 168 | 50 | 3 | 6·0 | 3·8 | 22.x.51 | | 168 | 50 | 3 | 6·0 | 7·5 | 27.i.52 |

* Cells showing clumped and sticky chromosomes.

*Key to Table IV.*

I. Time after treatment in hours.
II. Total number of cells in post-metaphase analysed.
III. Number and percentage of injured cells in post-metaphase.
IV. Percentage of cells in resting stage with micronuclei.
V. Date when animal was killed.

In the tumour 12 hours after treatment the rate of mitosis is still very low. The drastic reduction in the number of dividing cells is due to the high radiation dose which prevented cells at the end of the resting stage from entering mitosis. The delay in the developmental cycle may last 12 hours. This is indicated by the fact that in the 12-hour sample 6 cells with chromosome injuries were observed out of 27 in which the chromosome abnormality suggested that they were already in mitosis during irradiation. The frequency distribution of the injured cells observed in the various samples is a further indication that cell development has been retarded. While the highest proportion of injured cells is seen 12 hours after exposure to 300 r, the maximum damage was in the 48-hour tumour sample after 1000 r (Fig. 16). It is also interesting to report that the rate of mitosis was found to be very low in all samples. This fact explains the reduced growth-rate of the Walker carcinoma which occurs after treatment with 1000 r.

The data obtained in these experiments were sufficient to show that the frequency of injured cells decreases with the increase of time between irradiation and fixation of the tumour sample for examination ; thus 7 days after exposure to 1000 r only 6 per cent of the dividing cells were found to have chromosome injuries.

Special study has been made of the reaction of histiocytes in tumours irradiated with 1000 r. It was found that in all the samples under observation they make up only a relatively small fraction of the cell population which constitutes the tumour parenchyma. While there is, however, no histological alteration in the tumour proper (Fig. 17), a very definite change was noticed in the peripheral

FIG. 16.—The frequency distribution of injured tumour cells after various doses of X-ray and HN2 treatment. The reduction in the number of injured cells between 144 and 168 hours after HN2 is exaggerated owing to the fact that some cells have undergone two mitoses since treatment.

zone (connective tissue-capsule). This particular region in the 96-hour sample becomes very rich in histiocytes ; their increase is attributed to migration from the vascular system. In later samples the histological change (Fig. 18) resembles the initial stage of fibrous differentiation in the capsule of the Walker carcinoma described by Devik *et al.* (1950). The process, however, differs very much from the reaction which this carcinoma exhibits after HN2 administration. In the latter case histiocytes and fibroblasts increase throughout the whole tumour, which undergoes fibrosis. After a high dose of radiation " fibrosis " is restricted to the peripheral region, while the architecture of the tumour parenchyma remains unaltered. This explains why the resumption of tumour growth is more likely to occur after irradiation than after HN2 administration.

In view of these observations we may conclude therefore that not even the highest dose of X ray (1000 r) used in our experiments could bring about the same kind and same amount of histological changes as were seen after HN2 administration.

### DISCUSSION.

The most important difference observed in our experiments between the cytological effects of X rays and HN2 is the fact that the maximum number of injured cells is found in the tumour samples examined early after irradiation and later after nitrogen mustard treatment (Fig. 16). The true significance of this observation lies in the relationship which it has to the primary reactions induced by the two agents.

The view is now widely accepted that during irradiation ionisation takes place in the cell, followed immediately by the formation of free radicals of the water molecules (Lea, 1946). This event represents the first step in the complex reaction-chain which in time leads to a definite biological phenomenon. Similarly there is no substantial delay between administration of HN2 and its primary reaction in the organism. The rate of hydrolysis of nitrogen mustard is extremely high in a biological system, and it has been shown recently by Batemann, Klopp and Cromer (1951) that it remains " active " only for a few minutes after intravenous injection. This is the main reason which allows us to compare the cellular effects of X ray and HN2 in tumour samples taken at the same time after treatment and to determine the relative sensitivity of the various stages of the mitotic cycle.

The measure of sensitivity of a cell is the frequency of chromosome injuries observed at a given time. The interval between treatment and fixing the cell for analysis indicates the cell stage during treatment. Using this criterion the radiation sensitivity of the mitotic cycle has been determined in pollen grains of *Tradescantia* (Koller, 1946). If our present data are interpreted on the same criterion, then we must draw the conclusion that tumour cells of the Walker carcinoma are most sensitive to X rays at the end of the resting stage or beginning of prophase, while they are least sensitive to HN2 at this stage. The difference in sensitivity is illustrated in Fig. 16.

The same phenomenon was first reported by Ford (1949), who compared the frequencies of chromosome breaks in *Vicia* after X rays and HN2. Revell (1952) employed a new and critical method in the study of chromosome effects of several substances (mustards, epoxides, etc.) and came to the same conclusion. The high sensitivity of tumour cells in the intermitotic stage to HN2 may be a general property of cells. The fact that X ray and HN2 initiate their effects at different stages strongly suggests that their mode of action is different. Thus, for instance, we may assume that X rays act directly on definite linkages of the already synthesized polypeptide structure of the prophase chromosome thread, while a chemical agent affects the resting stage chromosome-filament either before or during the process of synthesis. This aspect of the chemical effects is discussed in more detail by Revell (1952).

The higher sensitivity of the resting stage to the chemical agents has another implication. The non-dividing (intermitotic or " resting ") cells are believed to be functionally the most active cells, and often referred to as cells in the " metabolic phase " (Hughes, 1952). It is not unlikely that the degeneration of many non-dividing tumour cells which we observed after HN2 administration is due to a gross disturbance in this metabolic phase. If it is found that chemical agents act preferentially on cells in the resting stage, a new approach may possibly be opened to the chemotherapy of cancer.

Because the functional or morphological characteristics of cells are determined in the resting stage it can be expected that cells of different types will show different degrees of response to HN2. Our experiment demonstrated such difference between tumour cells and histiocytes.

The study of the cytological behaviour of the Walker carcinoma under X ray and HN2 treatment has accordingly led to the conclusion that the mode of action of these agents differs, and there is other experimental evidence of different kinds to support this conclusion. By lowering the oxygen tension during irradiation, Baker and Sgourakis (1950) found a great reduction in the frequency of sex-linked lethals in *Drosophila*. No such reduction was observed when nitrogen mustard was used (Auerbach and Moser, 1951). In barley, mutations of particular genes were induced with a higher frequency by HN2 than was expected, indicating some specificity of action (Gustafsson and MacKey, 1948). No such phenomenon was demonstrated by ionizing radiation. The observation of Ford (1949) and Revell (1952), who found a higher breakage-frequency in particular chromosome and chromosome regions after HN2 administration, falls into the same category. Widner, Storer and Lushbaugh (1951) compared the effects of irradiation and HN2 on mitotic and intermitotic times in several normal and tumour tissues and found a significant difference which they attributed to the difference in the mode of action of these agents.

In view of the arguments presented above it would be a gross error to infer a similarity of the mode of action of HN2 and X rays, based on the similarity of some " end-products " (e.g., blistering of skin ; bleaching of pigmented hair, etc.). The latter are the result of a complex chain of reactions, which can be initiated by fundamentally different primary events. We believe this to be the case, and for that reason the objection is raised to labelling nitrogen mustard as a " radiomimetic " poison. Such an adjective, by implicitly emphasizing the resemblance of a series of biological phenomena which are " end-products," tends to obscure the important basic differences.

### SUMMARY.

1. Cytological analysis of the femoral bone marrow and implanted Walker carcinoma of the rat has shown that up to 24 hours after treatment, 200 roentgens produce the same amount of chromosome injuries as 1 mg. of nitrogen mustard (HN2) per kg. body weight when it is given by intraperitoneal injection.

2. The number of tumour cells with chromosome injuries decreases 12 hours after irradiation (200 r), and damaged cells are almost absent in tumours 72 hours after treatment.

3. When HN2 is administered the proportion of injured cells increases with time, and 144 hours after injection about 90 per cent of the dividing cells exhibit chromosome injury.

4. There is a rapid increase in the number of dividing histiocytes and fibroblasts 72 hours after HN2 administration and many tumour cells in the resting stage undergo degenerative changes. The histological organisation of the Walker carcinoma is greatly altered 7 days after injection.

5. Body weight is greatly reduced after HN2 administration, which indicates a toxic effect on the whole organism, which plays an important role in the reaction of the tumour cells and tissue.

6. Such systemic effects as are induced by whole body irradiation with 200

and 500 r do not increase cellular injury and fail to induce any histological transformation of the Walker carcinoma.

7. A single dose of irradiation (1000 r) can induce histological alteration, but of a type very different from that produced by HN2. The latter involves the entire tumour parenchyma of the Walker carcinoma, while the former is restricted to the connective-tissue capsule around the tumour.

8. The number of tumour cells and histiocytes showing chromosome injury after HN2 administration differs in the same tumour, indicating a different sensitivity in the two cell types to nitrogen mustard.

9. The significant difference found between the frequency of distribution of injured cells suggests that cells at the end of the resting stage are the most sensitive to X rays and least sensitive to HN2.

10. The difference in behaviour shown by the Walker carcinoma after irradiation and after nitrogen mustard treatment indicates that the primary basic reactions initiated by these agents are fundamentally different.

This investigation has been supported by grants to the Royal Cancer Hospital and Chester Beatty Research Institute from the British Empire Cancer Campaign, the Jane Coffin Childs Memorial Fund for Medical Research, the Anna Fuller Fund, and the National Cancer Institute of the National Institutes of Health, U.S. Public Health Service.

### REFERENCES.

AUERBACH, C.—(1950) *Pubbl. Staz. zool. Napoli*, Suppl. **22**, 1.
*Idem* AND MOSER, H.—(1951) *Experientia*, **7**, 341.
BAKER, W. K., AND SGOURAKIS, E.—(1950) *Proc. nat. Acad. Sci. Wash.*, **36**, 176.
BATEMANN, I. C., KLOPP, C. T., AND CROMER, I. K.—(1951) *Blood*, **6**, 26.
BOYLAND, E., CLEGG, J. W., KOLLER, P. C., RHODEN, E., AND WARWICK, O. H.—(1948) *Brit. J. Cancer*, **2**, 17.
DEVIK, F., ELSON, L. A., KOLLER, P. C., AND LAMERTON, L. F.—(1950) *Ibid.*, **4**, 298.
DUSTIN, P.—(1947) *Nature*, **159**, 794.
ELSON, L. A., AND LAMERTON, L. F.—(1949) *Brit. J. Cancer*, **3**, 414.
FORD, C. E.—(1949) *Hereditas, Lund.*, Suppl., p. 570.
GILMAN, A., AND PHILIPS, F. S.—(1946) *Science*, **103**, 409.
GUSTAFSSON, A., AND MACKEY, I.—(1948) *Hereditas, Lund.*, **34**, 371.
HUGHES, A. F. W.—(1952) 'The Mitotic Cycle.' London (Butterworth).
KARNOFSKY, D. E., GRAEF, I., AND SMITH, H. W.—(1948) *Amer. J. Path.*, **24**, 275.
KOLLER, P. C.—(1946) *Brit. J. Radiol*, **19**, 393.—(1947) *Brit. J. Cancer*, **1**, 38.—(1948) *Brit. J. Radiol.*, Suppl., p. 84.
LEA, D. E.—(1946) 'Actions of Radiations on Living Cells.' London (Cambridge Univ. Press).
PHILIPS, F. S.—(1950) *J. Pharmacol.*, **99**, 281.
REVELL, S.—(1952) Thesis submitted to University of London for degree of Ph.D.
WIDNER, W. R., STORER, B. J., AND LUSHBAUGH, C. C.—(1951) *Cancer Res.*, **11**, 877.